# Probing the Fucoxanthin-Chlorophyll *a*/*c*-Binding Proteins (FCPs) of the Marine Diatom *Fragilariopsis* sp. by Resonance Raman Spectroscopy

**DOI:** 10.1021/acs.jpcb.3c04346

**Published:** 2023-10-11

**Authors:** Charalampos Andreou, Charalampos Tselios, Aristos Ioannou, Constantinos Varotsis

**Affiliations:** Department of Chemical Engineering, Cyprus University of Technology, 95 Eirinis Str., Lemesos 3603, Cyprus

## Abstract

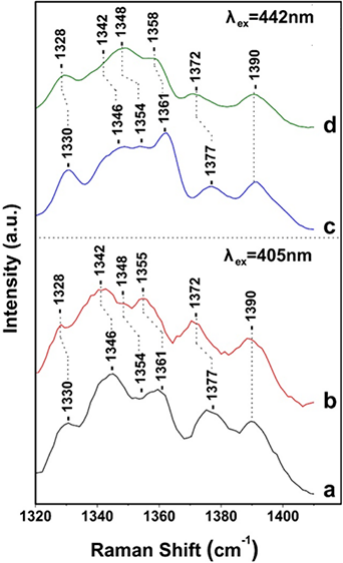

We report resonance Raman spectra of the light-harvesting fucoxanthin-chlorophyll *a*/*c*-binding proteins (FCPs) of marine diatom *Fragilariopsis* sp. The Raman shifts in the ^15^N-isotope-enriched diatom provide the first spectroscopic evidence for the characterization of the C_a_-N marker bands and, thus, of the penta- and hexacoordinated states of chlorophylls *a*/*c* in the FCPs. Under 405 and 442 nm Raman excitations, all of the marker bands of Chl *a*/*c* are observed and the isotope-based assignments provide new information concerning the structure of Chls *a*/*c* in the FCPs and their interactions with the protein environment. Therefore, the Raman spectrum at 405 nm originates from the π–π* transitions of Chl *a*/*c* and not from a different, non π–π* electronic transition, as previously reported (BBA Bioenergetics, 2010, 1797, 1647–1656). Based on the ^15^N isotope shifts of the C_a_-N and in conjunction with other marker bands, two distinct conformations of five- and six-coordinated Chl *a* and Chl *c* are observed. In addition, two keto carbonyls were observed at 1679 (strong H-bonded) and 1691 cm^–1^ (weak H-bonded) in both the 405 and 442 nm Raman spectra, respectively. Collectively, the results provide solid evidence of the nature of the vibrational modes of the active Chl *a*/*c* photosynthetic pigments in the FCPs.

## Introduction

Marine diatoms are involved in major photosynthetic biochemical cycles, in oxygenic photosynthesis and carbon fixation.^1−3^ They contain the light-harvesting pigment systems fucoxanthin-chlorophyll *a*/*c*-binding proteins (FCPs) to collect light energy in the blue-green region that is also available under water and transfer the trapped energy to the reaction centers where the primary electron transfer reactions convert energy into an electrochemical gradient.^4,5^ The photoacclimation strategy of species growing under variable light conditions enables the efficient regulation of photosystem structures to the amount of absorbed energy.^6^ Specific interactions of the pigment molecules in the protein environment and pigment–pigment interactions account for spectral and excitation energy transfer efficiency to chlorophyll *a* (Chl *a*).^7−9^ The structural differences in Chl *a* versus chlorophyll *c* (Chl *c*) lead to modified photophysical properties between the different types of macrocycles, which have been selected as the active pigments in marine photosynthesis.^10−12^

Resonance Raman spectroscopy has been applied extensively to characterize the structure of chlorophyll-containing proteins.^13−18^ The goal of these investigations was to determine the relationship between the protein control of the electronic and molecular structure at the chlorophylls and the physiological properties of the macromolecule. A satisfactory interpretational framework is not yet available despite a wealth of published data. The correct assignment of the marker bands of Chl *a*/*c* is of pivotal importance in understanding the molecular basis of their function since they display a wide range of frequencies depending on the protein properties. In order to fully utilize the potential of resonance Raman scattering, it is essential to assign the normal modes of Chl *a*/*c* in the FCPs. Raman spectroscopy has been applied in isolated FCPs from the centric diatom **Cyclotella meneghiniana** cells under frozen conditions (77K) with variable excitations from 406.7 to 476.5 nm.^15^ It was reported that under 441, 457.9, and 476.5 nm excitation, two distinct Chl *c*2 C13^1^ keto carbonyls were observed at 1675 (strong H-bonded) and at 1690 cm^–1^ (weak H-bonded) associated with the presence of two C_a_-N breathing modes of Chl *c*2 at 1355 and 1362 cm^–1^, indicating the presence of two conformers. Furthermore, it was reported that under 406 and 413 nm excitation, there is no evidence for the presence of the C_a_-N stretching modes and the C13^1^ keto carbonyl modes of Chl *c*2 and the C_a_-N breathing modes of Chl *a* are absent. It was suggested that the 406 and 413 nm excitation Raman spectra originate from a different, non π–π* electronic transition. This suggestion is overly simplistic but suggests that the origin of the Raman modes observed under 406 and 413 nm excitation is still unknown and raises the possibility that other vibrations despite the fact that they are not conjugated into the π-system of the macrocycle could gain Raman activity. Vibrational assignments of Chl *a*/*c* based on isotopically enriched ^15^N-FCPs are not available, yet. Resonance Raman spectra of ^15^N-enriched Chl *a*/*c* under 406 and 441 nm excitation are needed to establish a set of vibrational assignments of Chl *a*/*c* in the FCPs that will clarify their separate contributions in this energetic blue region.

In the work presented here, we have extended our work in diatoms and employed 406 and 441 nm Raman excitation to probe the Fx-Chl *a*/*c* pigment content in the FCPs from the marine diatom *Fragilariopsis* sp.^19^ The changes in ^15^N-containing Chl *a*/*c* bands in the FCP provide the first spectroscopic evidence for the characterization of the structural differences in Chl *a* versus Chl *c* that lead to altered photophysical and redox properties between the different classes of macrocycles, which are crucial for light harvesting and energy transfer processes. Based on the criteria that the Raman bands associated with large shifts upon ^15^N substitution are related to the C_a_-N stretching, where those that do not are related to the carbon–oxygen and carbon–carbon and/or the carbon–hydrogen, we have made the assignments of all Chl *a*/*c* modes. The 1361(−5) and 1354 (−5) cm^–1^ modes observed in the 405 and 442 nm excitation Raman spectra originate from the pyrrole breathing C_a_-N modes of two conformations of Chl *c* and those at 1377 (−5) and 1346 (−4) cm^–1^ to the C_a_-N of Chl *a*. There are also additional vibrational modes affected by the ^14^N to ^15^N substitution in the macrocycles of Chls *a*/*c*. Two keto carbonyls were observed at 1679 (strong H-bonded) and 1691 cm^–1^ (weak H-bonded) in both the 405 and 442 nm Raman spectra.

**Figure 1:**
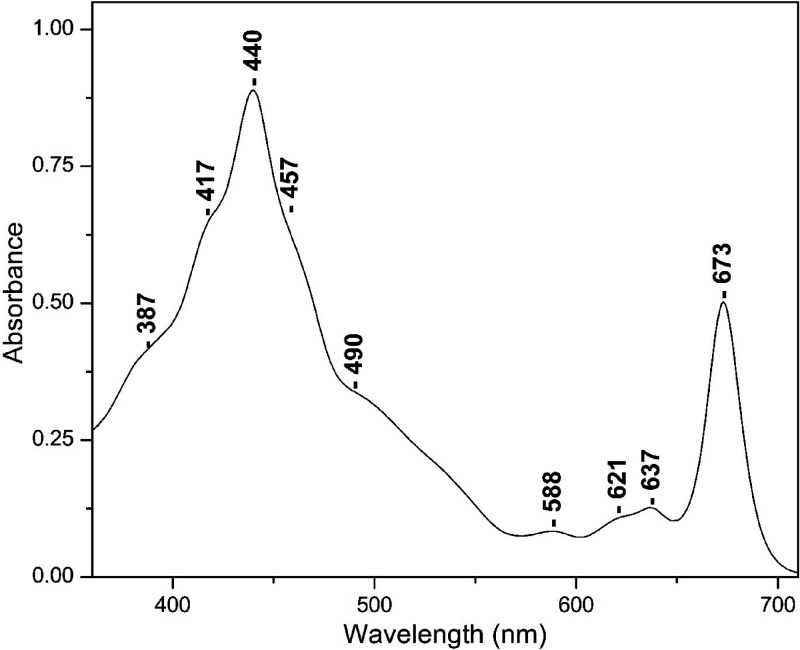
UV–vis absorption of FCP *Fragilariopsis* sp. at room temperature

**Figure 2:**
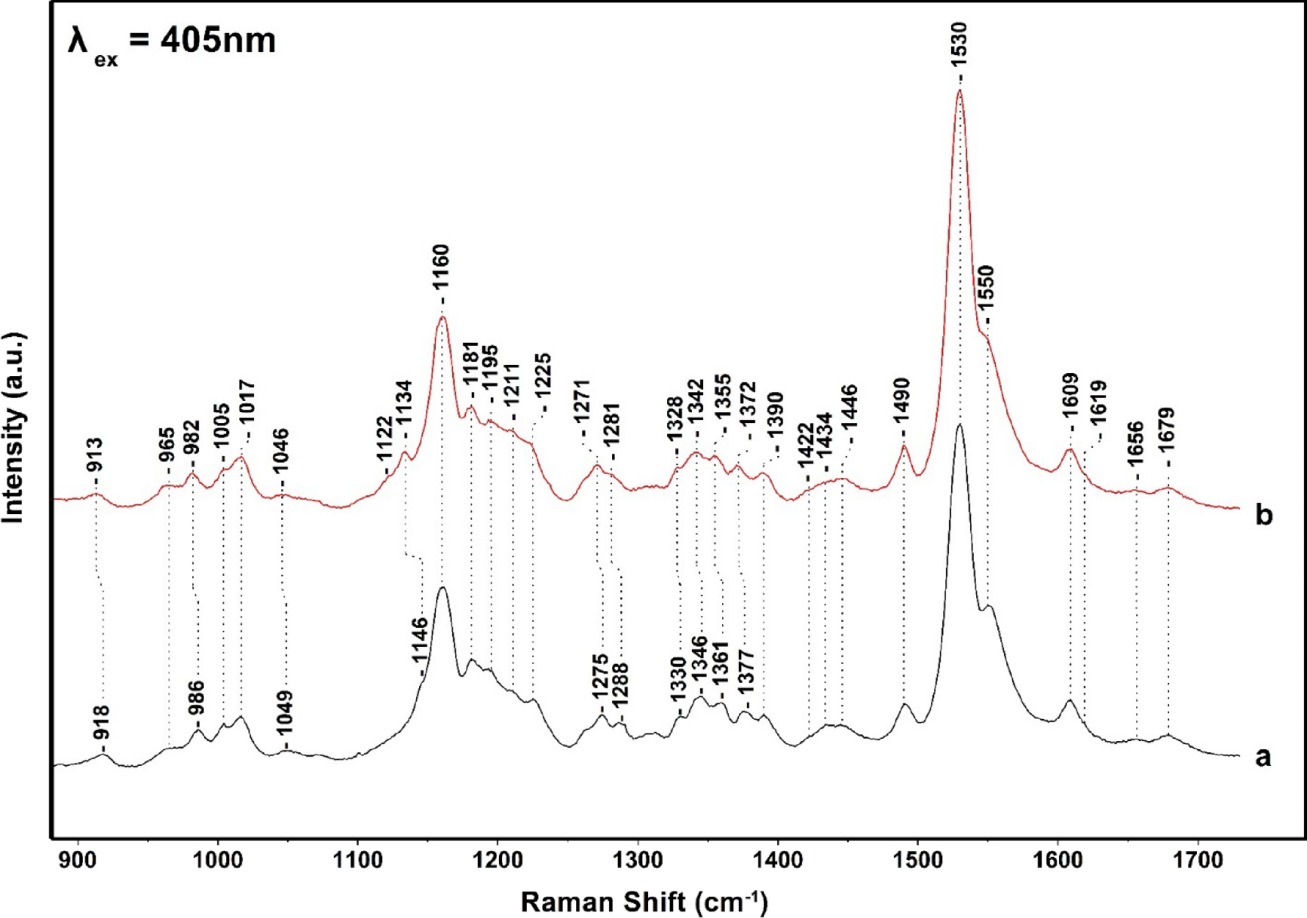
High-frequency 405 nm excitation resonance Raman spectra of NA (trace a) and ^15^N (trace b) FCP from *Fragilariopsis* sp. The 405 nm excitation laser beam was provided by a Coherent Laser, and the laser power incident on the sample was 10 mW. The total accumulation time for each measurement was 1 min. Every spectrum is the average of 10 measurements.

## Materials and Methods

The culture of *Fragilariopsis* sp. (CCAP 1029/24) was obtained from CCAP (Culture Collection of Algae and Protozoa). Cultures were grown in f/2-Si medium containing either Na^14^NO_3_ (NA) or ^15^NH_4_NO_3_ as nitrogen source, at 19 °C in a dark–light cycle (12h:12h) under white LED light. The light intensity used for the growth of the cell cultures was 150 μmol of photons m^–2^ s^–1^.

Cells from the *Fragilariopsis* sp. diatom were harvested by centrifugation (8000 rpm, 30 min, 4 °C), and the supernatant medium was removed. The cells were resuspended in 20 mM Tris and sonicated in an ice bath for 20 min. Thylakoids were then solubilized with the addition of β-1,4-dodecyl maltoside (β-DDM, 30 mg, 4 mM) in the resuspended cells while they were shaken for 20 min on ice. Separation of solubilized proteins was carried out on an ion exchange column (HiPrep Q HP 16/10, 20 mL) in 25 mM Tris, 2 mM KCl, and 0.4 mM β-DDM at pΗ 7.4 using a Shimadzu LC-20AD with a SPD-20A detector with two-wavelength detection. The samples were loaded in a column, and fractions were eluted using a gradient from 0 to 750 mM KCl in a buffer made of 25 mM Tris, 750 mM KCl, and 0.4 mM β-DDM, pΗ 7.4 at a flow rate of 3 mL/min. Fractions were pooled and concentrated using Amicon filtration devices with a cutoff of 10 kDa and characterized, spectroscopically and biochemically. The rest of the fractions were stored at −80 °C until further use. A 2:1:1 Chl*a*:Chl*c*:Fx stoichiometry was determined.

Absorption spectra were recorded with a Cary 60 UV–vis spectrometer (Agilent Technologies, USA). Raman data were collected by a confocal LabRAM (HORIBA Jobin Yvon, Kyoto, Japan) equipped with a CCD detector, 1800 grooves/mm grating, and an Olympus BX41 microscope. The spectral resolution was 5 cm^–1^. The 405 nm excitation was provided by a Coherent Laser, and the laser power incident on the sample was 10 mW. The 442 nm excitation was provided by a KIMMON He–Cd Laser, and the laser power incident on the sample was 10–15 mW. For the Raman measurements, the temperature of the samples was kept at −70 °C by using a Linkam cell with a liquid Nitrogen cryostat.

## Results and Discussion

Figure 1 shows the UV–vis spectrum of FCP from *Fragilariopsis* sp. at room temperature, at pH 8. The transitions at 440 (Soret), 621 (Q_*x*_), and 673 (Q_*y*_) nm are attributed to Chl *a*, and at 457 (Soret), 588 (Q_*x*_), and 637(Q_*y*_) nm to Chl *c*.^15,19^ The broad transition in the 500–560 nm range has been attributed to red Fxs, whereas blue Fx absorb in the 420–470 nm region. The pigment analysis (Andreou et al. ms in preparation) of the present FCP contains Chl *c*2 possessing a vinyl group at the 8-position conjugated to its porphyrin π-system.

One objective of this work is to identify the C_a_-N in Ch *a*/*c* by isotopic labeling of Ch *a*/*c* nitrogens (N). This way, the structure/function and coordination state of Chl *a*/*c* in the FCPs will be determined. In the Chl *a*/*c* macrocycles, rings I and II are aligned along the *y*-axis and modes associated with these rings should be enhanced using lines such as 405 nm excitation. Rings II and IV are aligned along the *x*-axis, and modes associated with these rings should be enhanced using lines such as 441 and/or 458 nm close to the B_*x*_ axis. Excitation within the B_*y*_ absorption band using the 406.7 nm line produces a spectrum dominated by totally symmetric Franck–Condon-active modes aligned along the *y*-axis of the macrocycle. The assignment of the Chl *a* vibrational modes is still controversial, whereas that of Chl *c* is still limited. The 406.7 nm RR spectra of Chl *a* in solution obtained by the group of Bocian^13^ and those recorded by 457 nm excitation by the group of Koyama^18^ in conjunction with ^15^N isotopes and normal coordinate analysis have been reported. Upon ^15^N substitution and normal coordinate analysis by the group of Koyama,^18^ the modes with the largest shift were νC_a_N(II)/νC_a_NC_a_(I) at 1138 (−11), νC_b_H(IV) at 1188 (−8), γC_b_H(IV) at 1220 (−5), νC_a_N(II, IV) at 1284 (−7), νC_a_N(IV) at 1342 (−6), and νC_a_N(IV) at 1376 (−5) cm^–1^. In addition, 1488 (νC_a_C_m_), 1534 (νC_b_C_b_)), 1550 (νC_b_C_b_ + C_a_C_m_(III)), 1604 (νC_a_C_m_ + C_b_C_b_)), and 1698 (νC9 = O) cm^–1^ have been identified.

Figure 2 shows the 405 nm excitation Raman spectrum of the FCP from ^14^N-containing (trace a) and ^15^N-containing FCP (trace b) at pH 8, 25 °C. The assıgnments of the peaks in trace a are secured vıa the ^15^N shifts shown ın trace b. The modes with a larger isotopic shift were associated with the C_a_-N stretching modes. In trace a, the peaks at 918, 1146, 1138, 1115, 1288, 1361, and 1377 cm^–1^ have contributions from C_a_N of Chl *a*/*c* and 1275 and 1346 cm^–1^ from C_m_H and C_a_N. The clearest indicator of Chl *c* is the C_a_-N mode at 1361 cm^–1^. Its frequency is similar to that observed for the oxidation state marker band in heme-containing protein with the same porphyrin ring structure.^20,21^ The five-coordinate Chl *a* complexes are characterized by modes in the frequency ranges 1605–1612, 1551–1555, and 1527–1529 cm^–1^ whereas those of six-coordinate are downshifted to 1596–1600, 1545, 1548, and 1518–1521 cm^–1^. We assign the peaks at 1609 and 1550 cm^–1^ to five-coordinated Chl *a* and the peaks at 1619 cm^–1^ to the vinyl of Chl *c*. For the assignments of 1550, 1656, and 1679 cm^–1^, see below. The peaks at 965, 1005, 1046, 1160, 1185, 1195, 1490, and 1530 cm^–1^ originate from Fx, in agreement with previous works.^19,22,23^

**Figure 3:**
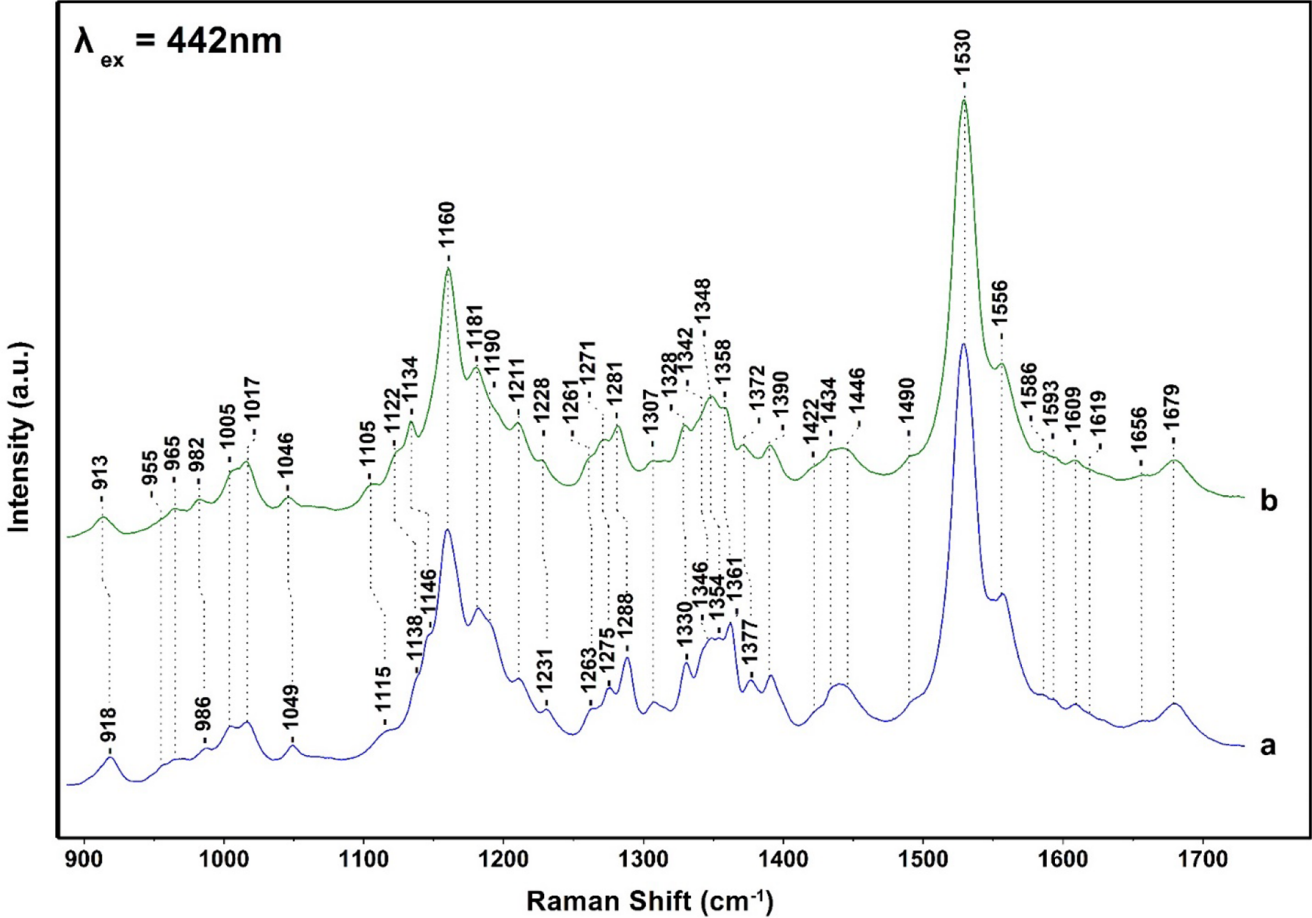
High-frequency 442 nm excitation resonance Raman spectra of NA (trace a) and ^15^N (trace b) FCP from *Fragilariopsis* sp. Trace a is the natural abundance (NA) FCP and trace b from cells grown in ^15^N. The 442 nm excitation laser beam was provided by KIMMON He–Cd Laser, and the laser power incident on the sample was 10 mW. The total accumulation time for each measurement was 1 min. Every spectrum is the average of 10 measurements.

Figure 3 shows the 442 nm excitation Raman spectra of the FCP from ^14^N-containing (trace a) and ^15^N-containing FCP (trace b) at pH 8, 25 °C. The assıgnments of the peaks ın trace a are secured vıa the ^15^N shıfts shown ın trace b and via the spectra obtained with 405 nm excitation. The most significant differences in the data presented in Figure 3 and those in Figure 2 are (1) the intensity increase of the 1362 (ν4 of Chl *c*) and decrease of 1346 (Chl *a*) modes and (2) the presence of the 1550 cm^–1^ mode observed in the 406 nm excitation and at 1556 cm^–1^ in the 442 nm excitation spectra. The latter indicates that 1556 cm^–1^ has contributions from the ν(C_a_C_m_+ C_b_-C_b_) of Chls *c* whereas the peak at 1550 cm^–1^ originates mostly from the ν(C_a_C_m_+ C_b_-C_b_) of Chl *a*. The distinct isotopic shift of the C_a_-N modes of Chl *a*/*c* in the ^15^N-labeled FCP confirm in conjunction with the presence of the 1608 and 1586/1593 cm^–1^ modes the presence of five- and six-coordinate Chl *a*. The magnitude of the isotopic shift agrees well with the calculated shift based on empirical normal coordinate analysis using the isotopes of Chl *a*.

**Figure 4:**
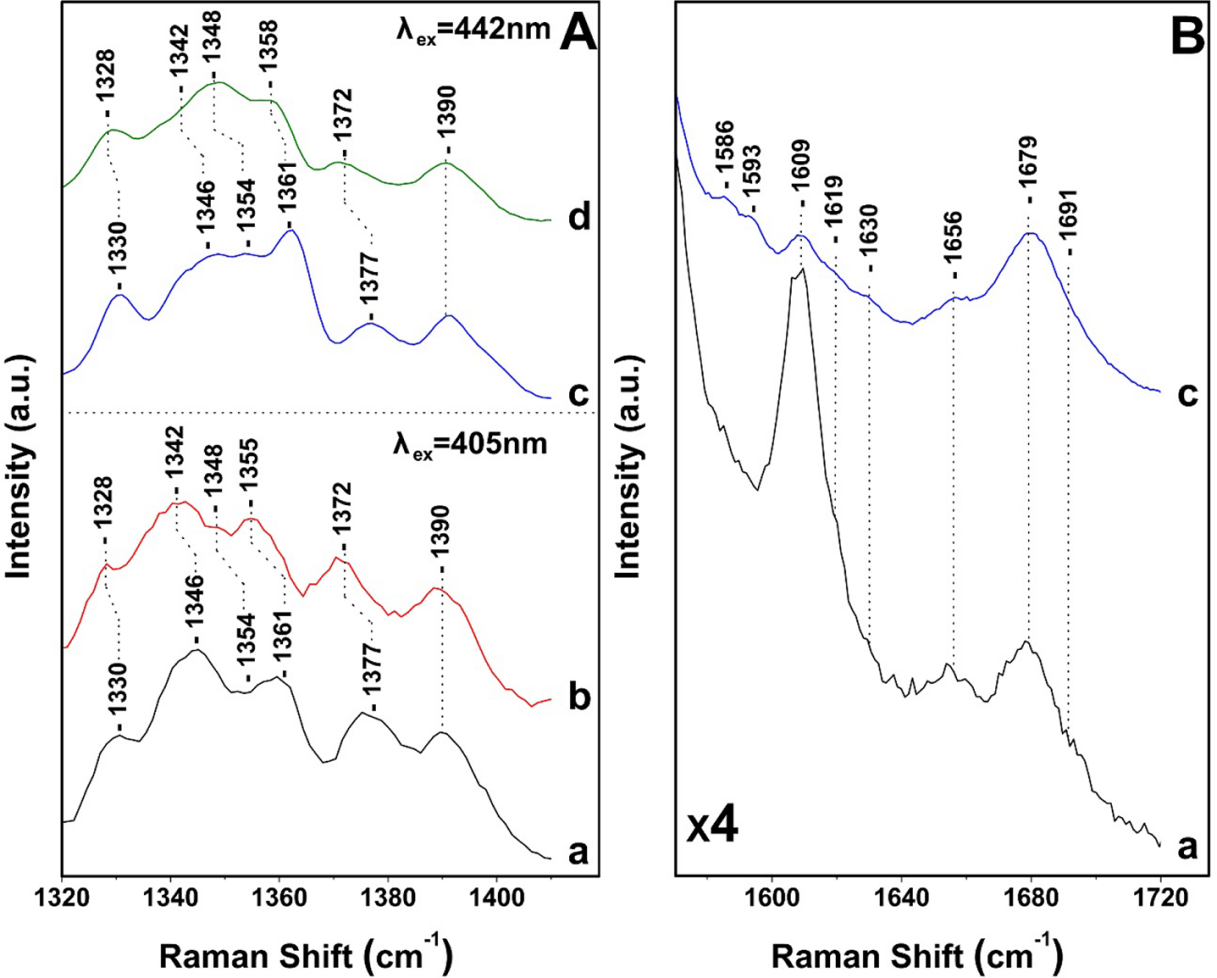
Expanded frequency range of the 405 and 442 nm Raman spectra presented in Figures 2 and 3. The spectra in (B) are ×4.

Expanded frequency and intensity of the 1320–1420 and 1580–1720 cm^–1^ regions of the data presented in Figures 2 and 3 are shown in Figure 4. In the 405 nm excitation spectra, there are three major isotope sensitive bands at 1377 (−5), 1346 (−4), and 1330 (−2) cm^–1^ that arise from five- and six-coordinated Chl *a* and two from Chl *c* at 1361 (−6) and 1354 (−6) cm^–1^ that originate from the two conformations of Chl *c*, as previously reported.^15^ However, the two conformations were observed only at 441, 457, and 476 nm excitations but not at 406 or 413 nm excitation. Interestingly, the same authors assigned the 1620 cm^–1^ observed in their 413 nm excitation spectra to the vinyl C3^1^=C3^2^ of Chl *c* but not the more intense C_a_N modes of Chl *c*. In addition, the C_a_N vibrations of Chl *a* were not reported. It should be mentioned that the Raman spectra in ref (15) were smoothed to recover the Raman spectra with a low signal-to-noise ratio (SNR). While this method is effective in reducing the noise signal, it has the undesirable effect of smoothing the underlying Raman features causing significant deviation from the “true” Raman signals. In the 1580–1720 cm^–1^ region, two bands at 1679 and 1691 cm^–1^ are observed and are attributed in agreement with previous assignments, to a medium-strength H-bonded and a weak H-bonded 13-keto C=O mode, respectively. The 1609 cm^–1^ peak arises from the methine bridges of Chls *a*/*c*, the frequency of which depends on the coordination state of the central Mg^2+^ ion and the 1630 cm^–1^ from the vinyl group. The coordination sensitive modes in Chl *a*/*c* indicate that most Chl *a*/*c* molecules are five-coordinated or there are certain six-coordinated Chls *a*/*c* molecules with weak axial ligands. The observation of the 1355/1361 cm^–1^ of Chl *c* clearly demonstrates that there is a subset of Chl *c* molecules that they experience changes in the core size.

**Figure 5:**
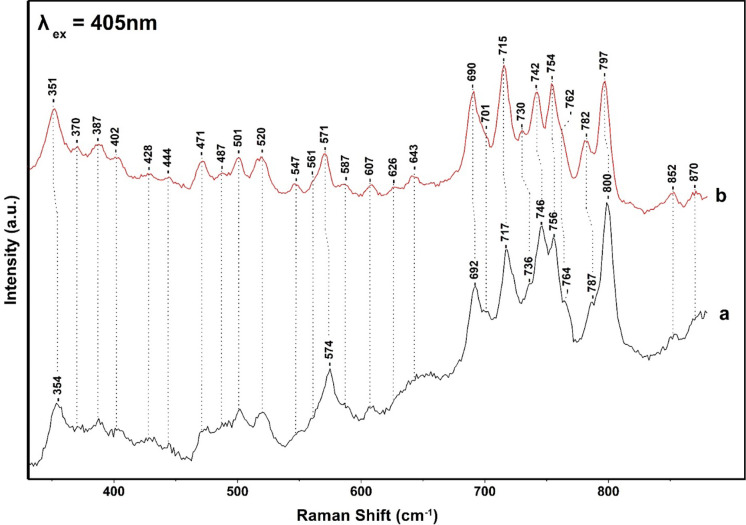
Low-frequency 405 nm excitation resonance Raman spectra of NA (trace a) and ^15^N (trace b) FCP from *Fragilariopsis* sp. The 405 nm excitation laser beam was provided by a Coherent Laser and the laser power incident on the sample was 10 mW. The total accumulation time for each measurement was 1 min. Every spectrum is the average of 10 measurements.

**Figure 6:**
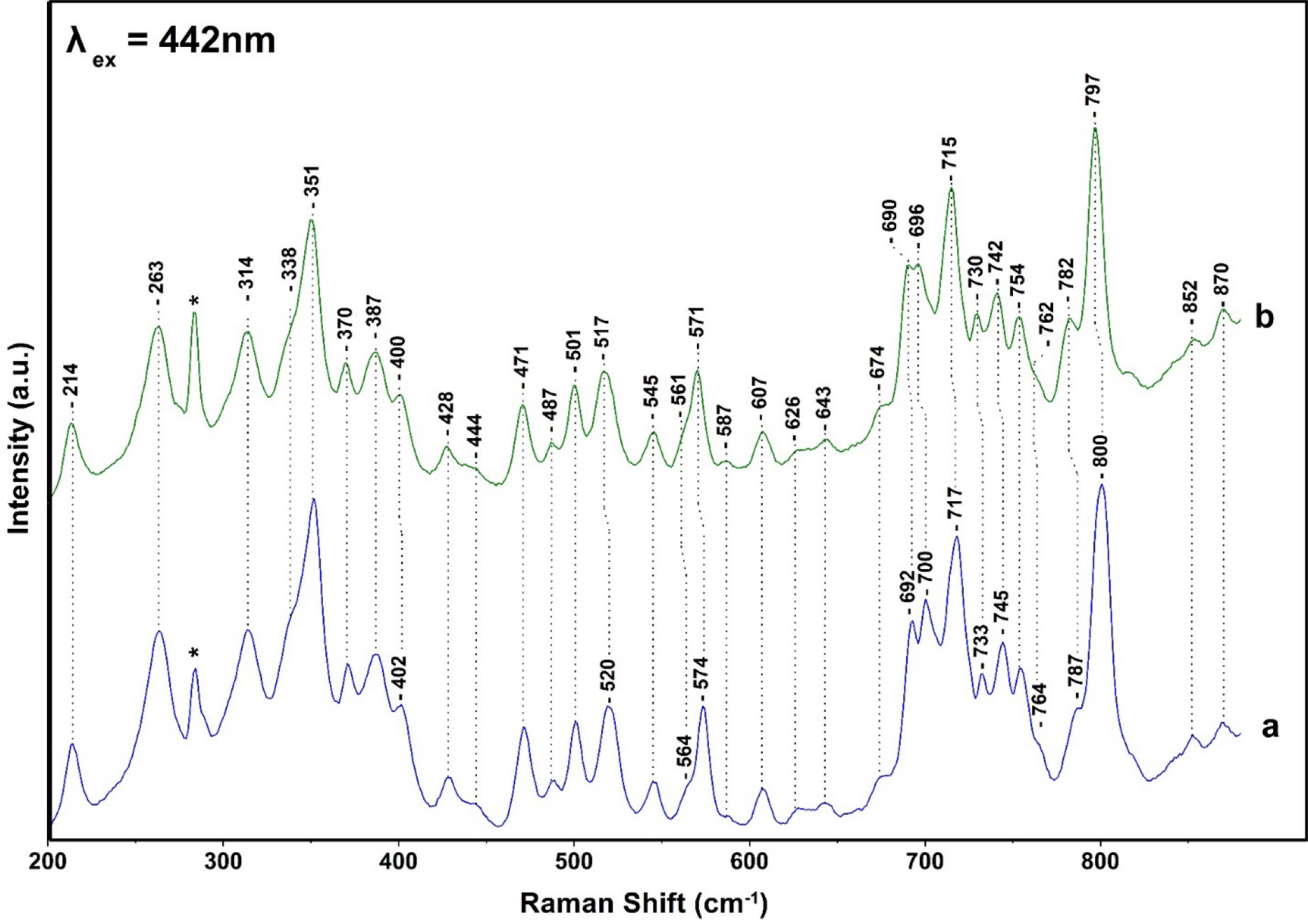
Low-frequency 442 nm excitation resonance Raman spectra of NA (trace a) and ^15^N (trace b) FCP from *Fragilariopsis* sp. FCP from *Fragilariopsis* sp. Trace a is the NA FCP and trace b from cells grown in ^15^N. The 442 nm excitation laser beam was provided by KIMMON He–Cd Laser, and the laser power incident on the sample was 10 mW. The total accumulation time for each measurement was 1 min. Every spectrum is the average of 10 measurements.

The high-frequency skeletal modes are understood and have been discussed several times with respect to their function as marker bands for oxidation/coordination/spin state sensitivity. Below the ν4 bands in the 1300–1400 cm^–1^ region, assignments of both Chl *a* and Chl *c* have not been reported in the past and are uncertain because of spectral congestion, which is due to activation of substituent modes, the Chl *a*/*c* out-of-plane modes, the Mg-ligand modes of Chl *a*/*c*, and the in-plane skeletal modes.^13^ Based on previous reports on Chl *a*, MgOEP, and heme proteins, we tentatively assign the low-frequency modes shown in Figures 5 and 6. Figure 5 shows the low-frequency region of the spectra presented in Figure 2, and those of the spectra presented in Figure 6 are those of the spectra presented in Figure 3. The spectra presented in Figure 5 revealed ^15^N isotope bands at 574 cm^–1^ (−3) and at 692 (−2), 717 (−2), 736 (−6), 746 (−4), 756 (−2), 764 (−20), and 800 (−3). In Figure 6, the ^15^N isotope shifts of a number of bands at 520 (−3), 574 (−3), 700 (−4), 717 (−2), 733 (−3), 745 (−3), 787 (−5), and 800 (−3) are depicted. The crystal structure of FCP has demonstrated that Chl *a*/*c* are coordinated by His, Tyr, and H_2_O axial ligands. Based on the vibrational analysis of heme- and chlorin-based assignments, we tentatively assign the 214 cm^–1^ mode presented in Figure 6 to the Mg–N symmetrical stretch (His of Chl *c*) of the five-coordinated Chl *c* and the 338 cm^–1^ mode to the asymmetrical stretch of the six-coordinated Chl *c*. The following tentative analysis of the ^15^N-sensitive bands is based on the normal coordinate analysis of Ni-OEP.^24^ The 263 (ν9) and 351 (ν8) cm^–1^ modes are pyrrole-substituent bending vibrations. In the 351–400 cm^–1^ region, there are unique bending vibrations related to the C–C–C of the acrylate substituents of Chl *c*. There are candidate RR bands for the acrylate moiety in Chl *c*, at 351–370 and 387–400, modulated by different orientations relative to the porphyrin plane of Chl *c*. These bands are insensitive to isotope labeling on the ring. In the 471 to 607 cm^–1^ region, there are three ^15^N-sensitive bands at 520 (−3), 564 (−3), and 574 (−3) cm^–1^ that may represent Mg-ligand vibrations. In the 690 to 800 cm^–1^ range, there are RR bands sensitive to ^15^N that may have contributions from both Chl *c* and Chl *a*. The vibrations are assigned to ν7 (700 cm^–1^), γ11(717 cm^–1^), γ5 (733 cm^–1^), ν15 (745 cm^–1^), and ν6 (800 cm^–1^).

The structures of the Chl *a*/*c* molecules in the lowest excited singlet and triplet states play key roles in the primary processes of photosynthesis. The studies reported herein for Chl *a*/*c* in the FCP provide insight in the key structural element that distinguishes Chl *a* from Chl *c*. Comparison of the FCP of *Fragilariopsis* sp. to those from other marine diatoms with known crystal structures may provide the means to identify conserved structural features that can be assumed to be involved in basic functions to different classes of FCPs. In contrast, dissimilarities between these FCPs are likely to be involved in the fine-tuning to specific needs demanded by differences in the local protein environment of the FCPs. Furthermore, with the identification of the major coordination marker bands of Chl *a*/*c,* the changes in the electronic and molecular structures of the macrocycles upon singlet and triplet excitation can also be obtained with more certainty.
